# Beneficial effects of SGLT2 inhibitor on metabolic inflexibility and visceral fat amount in animal model of obese type 2 diabetes

**DOI:** 10.1016/j.heliyon.2022.e11012

**Published:** 2022-10-10

**Authors:** Kento Hara, Yusuke Sakai, Yuji Tajiri, Masatoshi Nomura

**Affiliations:** a Division of Endocrinology and Metabolism, Kurume University School of Medicine, Kurume, Japan; b Institute of Animal Experimentation, Kurume University School of Medicine, Kurume, Japan; c Diabetes Center, Kurume University Medical Center, Kurume, Japan

**Keywords:** Obesity, Type 2 diabetes mellitus, Metabolic inflexibility, SGLT2 inhibitor, Fat oxidation

## Abstract

**Background:**

Obesity and type 2 diabetes mellitus (T2DM) are often accompanied with a disrupted diurnal rhythm of eating and sustained anabolic state, leading to metabolic inflexibility. In the present study, we plan to investigate effects of a sodium glucose co-transporter 2 (SGLT2) inhibitor, canagliflozin (CANA), on such a metabolic inflexibility, especially on fat metabolism, in the obese type 2 diabetic rats.

**Materials and methods:**

Five-week-old male SDT (Spontaneously Diabetic Torii) fatty rats as a model of obesity and T2DM and Sprague-Dawley (SD) rats were treated by either CANA (10 mg/kg) or saline (vehicle) orally for 14 days. Then, after the measurement of respiratory quotient (RQ) and visceral and subcutaneous fat volumes, rats were euthanized and blood and tissue samples were collected.

**Results:**

The treatment by CANA significantly enhanced β-ketone concentration in the blood during light period in the SDT fatty rats with no effect on blood glucose concentration. The CANA treatment significantly reduced visceral fat volume in the SDT fatty rats. A diurnal rhythm of RQ was severely disrupted and persistently high throughout the day in the vehicle-treated SDT fatty rats. By the administration of CANA clearly restored the disrupted diurnal rhythm of RQ with a revival of the nadir during light period. Quantitative real-time RT-PCR revealed a significant increase of AMP-activated protein kinase and decrease of acetyl-CoA carboxylase-1 expression in the liver, and a significant increase of hormone sensitive lipase and uncoupling protein-2 expression in the white adipose tissue by the treatment of CANA in the SDT fatty rats.

**Conclusion:**

CANA as a SGLT2i reduced visceral fat amount via the enhancement of fat oxidation during the light period, leading to an amelioration of metabolic inflexibility in an obese diabetic model. A novel mechanism of CANA prompts the possibility that this new class of anti-diabetic agent could be a promising anti-obesity agent as well.

## Highlights

• SGLT2i reduced visceral fat independent of its effect on urinary glucose excretion.
• SGLT2i increased blood ketone concentration during light period.
• Concomitantly, RQ during light period was decreased, reviving a diurnal RQ rhythm.
• Changes in relevant molecules were observed towards fat oxidation.
• SGLT2i effectively corrected metabolic inflexibility in an obese diabetic model.

## Graphical abstract

**Figure:**
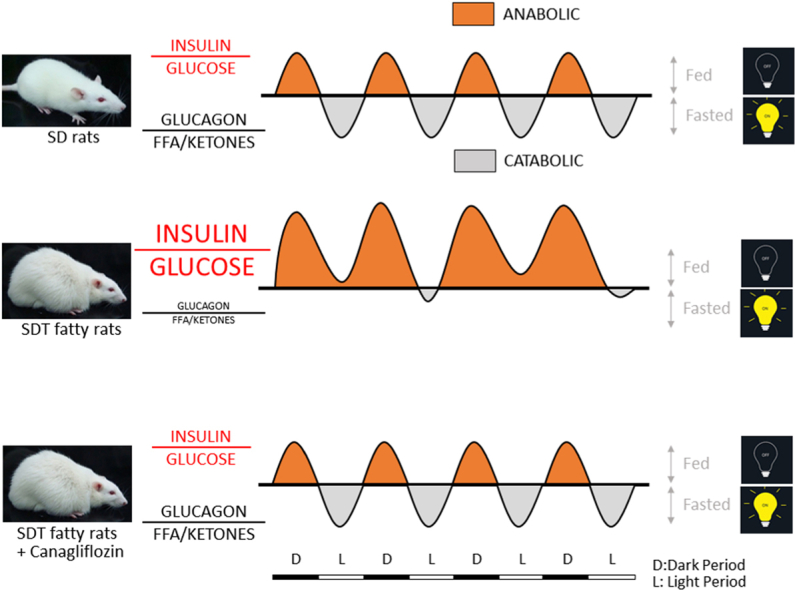

## 1 Introduction

Obesity is a serious burden and the number of obese patients is explosively increasing not only in the industrialized nations but also in developing countries. Obesity is a risk factor for lifestyle-related disease such as type 2 diabetes (T2DM), hypertension and dyslipidemia. These disorders are closely related to serious atherosclerotic complications such as cardiovascular diseases. Obesity per se is a cause of physical impediment such as osteoarthritis of knee or hip joint and sleep apnea syndrome, both of which are expected to be effectively recovered by a moderate reduction of current body weight. Therefore, the development of therapeutic strategies for the reduction of body weight has long been awaited.

Obesity is often associated with physical inactivity and disrupted life rhythms, including binge and night eating [1], which makes the treatment of obesity more complicated and weight reduction less attainable. In this context, a concept of ‘metabolic inflexibility’ is proposed in obese individuals. In lean subjects, fuel resource during fasting state is mainly free fatty acid (FFA) based on respiratory quotient (RQ) around 0.82, while during post-prandial state fuel resource shifts mainly to glucose with an RQ around 1.00, indicating that glucose is virtually the only energy source. In contrast, in the obese subjects the RQ of 0.9 in the fasting state does not change at all during post-prandial state, suggesting no shift of fuel resource and metabolic inflexibility [2]. In obesity or insulin-treated type 2 diabetes, the patient suffers from sustained anabolic state driven by the excess of caloric intake or lack of exercise and reinforced by insulin or insulin-enhancing therapies to lower circulating glucose, leading to a loss of metabolic rhythm and metabolic inflexibility [3].

Sodium-glucose co-transporter 2 inhibitor (SGLT2i) was first approved in 2014 in Japan. SGLT2i blocks SGLT2 located in the proximal tubules of the kidney and enhances the excretion of glucose into urine, thus contributes to the amelioration of blood glucose levels as well as the reduction of body weight. Because its glucose-lowering effect is not operated by either insulin secretion or insulin sensitivity, the concern for hypoglycemia is scarce by a sole administration of this agent for the treatment of type 2 diabetes (T2DM). It has been reported that SGLT2i reduces body weight, predominantly by reducing fat mass composed of either visceral or subcutaneous adipose tissue in T2DM patients [4]. Although caloric loss into urine is thought to be the major factor to explain the weight reduction by this agent, it is feasible that some other mechanisms exist considering the comparable amount of weight loss observed in patients with or without renal impairment [5, 6]. This distinct effect on weight loss from glucose-lowering effect, which we recently reported in T2DM patients [7], prompted us to explore some novel mechanisms of this agent independent of urinary glucose excretion. Given that effects of SGLT2i mimics catabolic state such as the increase of fat oxidation, ketone production and glucagon/insulin ratio, the administration of SGLT2i could be expected to enhance catabolic response during night and to restore dysregulated metabolic rhythm in obesity or T2DM patients.

In the present study, we investigated effects of SGLT2i, canagliflozin, on energy metabolism including RQ and fat oxidation as well as body composition in animal model of obesity and T2DM.

## 2 Materials and Methods

### 2.1 Animals

Male Sprague–Dawley (SD) rats and SDT (Spontaneously Diabetic Torii) fatty (SDT-fa/fa) rats (Jcl:SD and SDT/Jcl, CLEA Co Ltd., Osaka, Japan) were used in this study. The SDT fatty rat was established as the congenic obesity-related type 2 diabetes model by introducing the fa allele of the Zucker Fatty rat into the genome of the original SDT rat [8]. The animals were housed in a controlled room (temperature 25 ± 2 °C, humidity 60 ± 10%) under 12 h light–dark cycle (light on 0700–1900) with *ad libitum* access to control chow diet (CD; 10 kcal% fat, produced by Research Diets, Inc., New Brunswick, NJ, USA: open source diet code D12450B) and water.

All the experiments were performed in accordance with protocols approved by the Kurume University Animal Experiment Committee, based on the National Research Council's Guide for the Care and Use of Laboratory Animals (1996).

### 2.2 Experimental protocol

Canagliflozin (CANA) was synthesized by Mitsubishi Tanabe Pharma Corporation, Medicinal Chemistry Laboratory (Toda, Saitama, Japan) [9]. CANA was formulated in 0.5% hydroxypropyl methylcellulose [10]. Either SD or SDT fatty rats at five weeks old were individually housed in ordinary clear plastic TPX® cages (W27 cm × D43 cm × H20 cm) with paper bedding, and oral administration of either CANA (10 mg/kg) or vehicle (the same amount of saline) using gavage was performed in every morning (0900–1000) for 14 days (n = 8 in each group). At day 12, they were moved individually into specially designed acrylic metabolic chambers equipped with gas analysis system (ARCO system, Chiba) to measure respiratory gas for the calculation of respiratory quotient (RQ).

### 2.3 Measurement of body weight, food intake, and fat distribution

Body weight was measured every morning, and food intake and the amount of drinking were measured at day 1 and day 14. A saucer had been equipped beneath each feeder, and food spill was collected and measured. Food intake was then calculated subtracting this spilt food from the intake value. All the measurements were conducted before oral administrations of either CANA or vehicle.

Visceral and subcutaneous fat volumes (mm^3^) were measured in the afternoon (1400) at day 1, 7 and 14 using in vivo micro-computed tomography (R_mCT2, Rigaku Co., Tokyo, Japan) under imaging conditions of FOV73 (φ 73 mm × H57 mm), 90 kV tube voltage and 160 μA tube current. Rats were anesthetized with 3% isoflurane (Wako Pure Chemical Industries, Ltd., Osaka, Japan) and placed supine in the machine, and serial 4 mm scans were performed from the anterior to the posterior aspect of lumbar vertebra 4. Fat analysis software (Rigaku Co., Tokyo, Japan) estimated the volumes of adipose tissue, bone, air and the remainder on the basis of their different X-ray densities, and distinguished visceral and subcutaneous fat tissues by detecting the abdominal muscle layers.

Blood sugar during the light period (1400–1600) and β-ketone concentration during the dark (2000) and light (1400–1600) period were measured in SDT fatty rats at day 14 by tail snipping using handheld instrument (Precision Xceed; Abbott Co., Chiba, Japan).

Twenty 4-h urine samples were collected for the measurement of urine volume (day 1–2 and 14–15) and urine sugar (day 14–15) in the 4 groups of rats.

### 2.4 Respiratory gas analysis

The instruments and software used for the measurement of oxygen consumption and respiratory quotient of rats were obtained from ARCO Systems (Chiba, Japan). The system consisted of eight acrylic metabolic chambers as mentioned above, a mass spectrometer (model ARCO-2000) and a gas sampler (model ARCO-2000-GS10). Each metabolic chamber had a sedentary room (752 cm^2^ floor and 20 cm in height). Room air was pumped through the chambers at a rate of 2.0 L/min. The air from each chamber was sampled for 15 s. During the last 5 s, VO_2_ and VCO_2_ concentration were measured, and RQ (VCO_2_/VO_2_) was calculated. The respiratory data for each chamber were obtained every 5 min, and all the data for 12 h during light or dark period at day 13 and 14 were collected and the mean values were calculated from each 144 samplings, respectively. After respiratory gas measurements were performed, all the measurements including body weight, food consumption, blood sugar and ketone, urine collection and micro-computed tomography.

### 2.5 Quantitative real-time PCR

After all measurements were finished at day 15, rats were euthanized at 14:00 under the anesthesia with 3% isoflurane, and liver and white adipose tissue samples were collected for quantitative RT-PCR to measure fat oxidation-related enzyme. RNA was isolated using RNA-Bee (Cosmo Bio, Tokyo, Japan), and 5 mg of total RNA was reverse-transcribed to cDNA using a kit from Invitrogen (Carlsbad, CA, USA). SYBR green-based real-time quantitative PCR of cDNA templates was performed using StepOnePlus (Applied Biosystems, Foster City, CA, USA). The PCR cycling conditions were 10 min at 95 °C followed by 40 cycles of 30 s at 95 °C, 30 s at 53–64 °C, and 30 s at 72 °C. The results were calculated as the expression of the target gene relative to the expression of the glyceraldehyde 3-phosphate dehydrogenase (GAPDH) gene. Primers for RT-PCR are listed in Table 1.

**Table 1:** Designed primers.

Gene	Reverse primer (5′-3′)	Forward primer (3′-5′)
Acetyl-CoA Carboxylase-1 (ACC-1)	AGGTGCTCAAGTTTGGTGCT	TGGGTCGATCACAACCCAAG
AMP-activated protein kinase (AMPK)	TTATGCAGCACCGGAGGTC	AGAACACACCCCCTCGGAT
Hormone Sensitive Lipase (HSL)	CCCATACCCCATTGCCTG	CTGCCTCAGACACACTCCTG
Uncoupling Protein-1 (UCP-1)	CCTCTCCGGTGGATGTGGTAAA	CGCAGAAAAGAAGGCGCAAA
Uncoupling Protein-2 (UCP-2)	GGCGGTGGTCGGAGATA	GGCAGAAGTGAAGTGGCAAGGG
GAPDH	GTGCCAGCCTCGTCTCATAG	AAGAGAAGGCAGCCCTGGTA

### 2.6 Statistical analysis

All tests were performed using JMP Pro Ver. 15 (SAS Institute Inc., USA). Data are presented as the means ± SEM. Student's t-test was used for the comparisons between vehicle-treated and CANA-treated group and between SD rats and SDT fatty rats under the same treatment. Paired t-test was used for the comparisons between blood ketone concentrations or RQs during light and dark period. A P-value < 0.05 was considered to be statistically significant.

## 3 Results

### 3.1 Changes in food and water consumption, urine volume and urine sugar after the administration of canagliflozin for 14 days

As shown in Table 2, food intake was significantly increased in SD rat by the administration of CANA for 14 days. In SDT fatty rats, food intake was markedly greater compared to that in SD rats whereas CANA treatment didn't enhance it significantly at either day 1 or day 14. By the administration of CANA, drinking volume and urine volume were markedly enhanced both in SD and SDT fatty rats at either day 1 or day 14.

**Table 2:** Food intake, drinking and urine volume in SD and SDT fatty rats either treated by canagliflozin or not.

		SD + vehicle	SD + CANA	SDT fatty + vehicle	SDT fatty + CANA
food intake (g/day)	day 1	7.8 ± 3.4	9.5 ± 1.9	21.1 ± 1.8^##^	23.0 ± 2.4^##^
	day 14	7.8 ± 2.8	18.5 ± 0.9∗	26.2 ± 3.6^##^	32.5 ± 3.9^#^
drinking volume (mL/day)	day 1	8.5 ± 2.5	19.0 ± 2.1∗	25.1 ± 8.6	42.8 ± 12.3∗
	day 14	22.3 ± 3.9	56.0 ± 3.7∗∗	33.4 ± 5.4	54.3 ± 4.5∗∗
urine volume (mL/day)	day 1	5.1 ± 0.7	13.8 ± 1.4∗∗	14.7 ± 5.2	24.2 ± 1.8^##,^ ∗∗
	day 14	10.6 ± 3.2	37.0 ± 2.0∗∗	21.0 ± 2.9^#^	46.8 ± 5.3∗∗
urine sugar (g/day)	day 14	0	3.36 ± 0.47∗∗	1.58 ± 0.65^##^	3.40 ± 0.18∗

Data are presented as means ± SEM. SD; SD rats. SDT fatty; SDT fatty rats. CANA; canagliflozin. ∗P < 0.05, ∗∗P < 0.01 vs. vehicle-treated data. #p < 0.05, ##p < 0.01 vs. SD rats.

The increase of urine sugar was brought about even in SD rats by CANA treatment for 14 days. In SDT fatty rats, however, no more significant increase of urine sugar was observed by CANA treatment.

### 3.2 Blood glucose and β-ketone concentration after the administration of canagliflozin for 14 days

**Figure 1:**
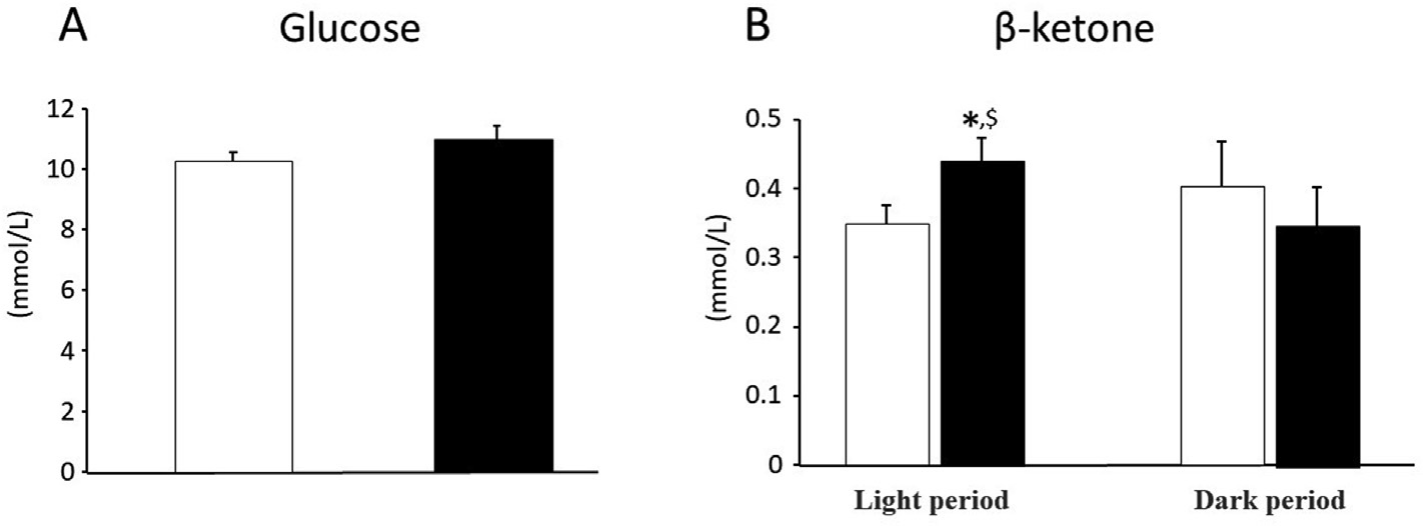
Blood glucose (A) and β-ketone concentration (B) after the administration of canagliflozin in SDT fatty rats for 14 days. Open bars denote data in vehicle-treated group and black bars those in CANA-treated group. Data are presented as means ± S.E.M. ∗P < 0.05 vs. vehicle-treated group. ^$^P < 0.05 vs. dark period.

As shown in Figure 1, the administration of CANA for 14 days didn't affect blood glucose concentration at all in the SDT fatty rats (10.2 ± 0.4 mmol/L by vehicle vs. 10.9 ± 0.6 mmol/L by CANA). In contrast, 14-day treatment by CANA significantly (P < 0.05) enhanced β-ketone concentration in the blood during light period (0.44 ± 0.03 mmol/L) compared to that during dark period (0.34 ± 0.06 mmol/L), while no obvious effect was observed in vehicle-treated SDT fatty rats (0.35 ± 0.03 mmol/L during light period vs. 0.4 ± 0.07 mmol/L during dark period).

### 3.3 Changes in body weight and the percentage of subcutaneous or visceral fat volume after the administration of canagliflozin for 14 days

Body weight, subcutaneous or visceral fat volume was not affected by 14-day treatment by CANA in SD-rats. In SDT fatty rats, marked increases in subcutaneous and visceral fat volume were observed either at day 1 or day 14 compared to SD rats although there was no significant difference in body weight between the two groups. CANA treatment for 14 days significantly reduced visceral fat volume in SDT fatty rats (Table 3).

**Table 3:** Changes in body weight and the percentage of subcutaneous or visceral fat volume for 14 days of treatment.

		SD + vehicle	SD + CANA	SDT fatty + vehicle	SDT fatty + CANA
body weight (g)	day 1	125 ± 10	128 ± 8	135 ± 5	133 ± 12
	day 7	184 ± 7	177 ± 12	195 ± 3	189 ± 5
	day 14	241 ± 6	236 ± 13	250 ± 2	247 ± 7
subcutaneous fat (%)	day 1	3.2 ± 0.3	3.5 ± 0.5	23.1 ± 1.1^##^	21.0 ± 1.0^##^
	day 7	4.5 ± 0.3	2.6 ± 1.0	22.9 ± 1.4^##^	24.1 ± 1.5^##^
	day 14	3.1 ± 0.7	6.1 ± 2.6	27.8 ± 1.7^##^	24.0 ± 2.0^##^
visceral fat (%)	day 1	2.0 ± 0.5	2.2 ± 0.4	21.7 ± 1.8^##^	20.5 ± 1.5^##^
	day 7	5.6 ± 1.2	4.2 ± 1.2	29.7 ± 1.7^##^	26.6 ± 1.8^##^
	day 14	5.4 ± 1.2	9.9 ± 2.7	35.1 ± 1.1^##^	31.0 ± 1.4^##,^ ∗

Data are presented as means ± SEM. SD; SD rats. SDT fatty; SDT fatty rats. CANA; canagliflozin.

∗P < 0.05 vs. vehicle-treated data. ##p < 0.01 vs. SD rats.

### 3.4 Respiratory quotient after the administration of canagliflozin for 14 days

**Figure 2:**
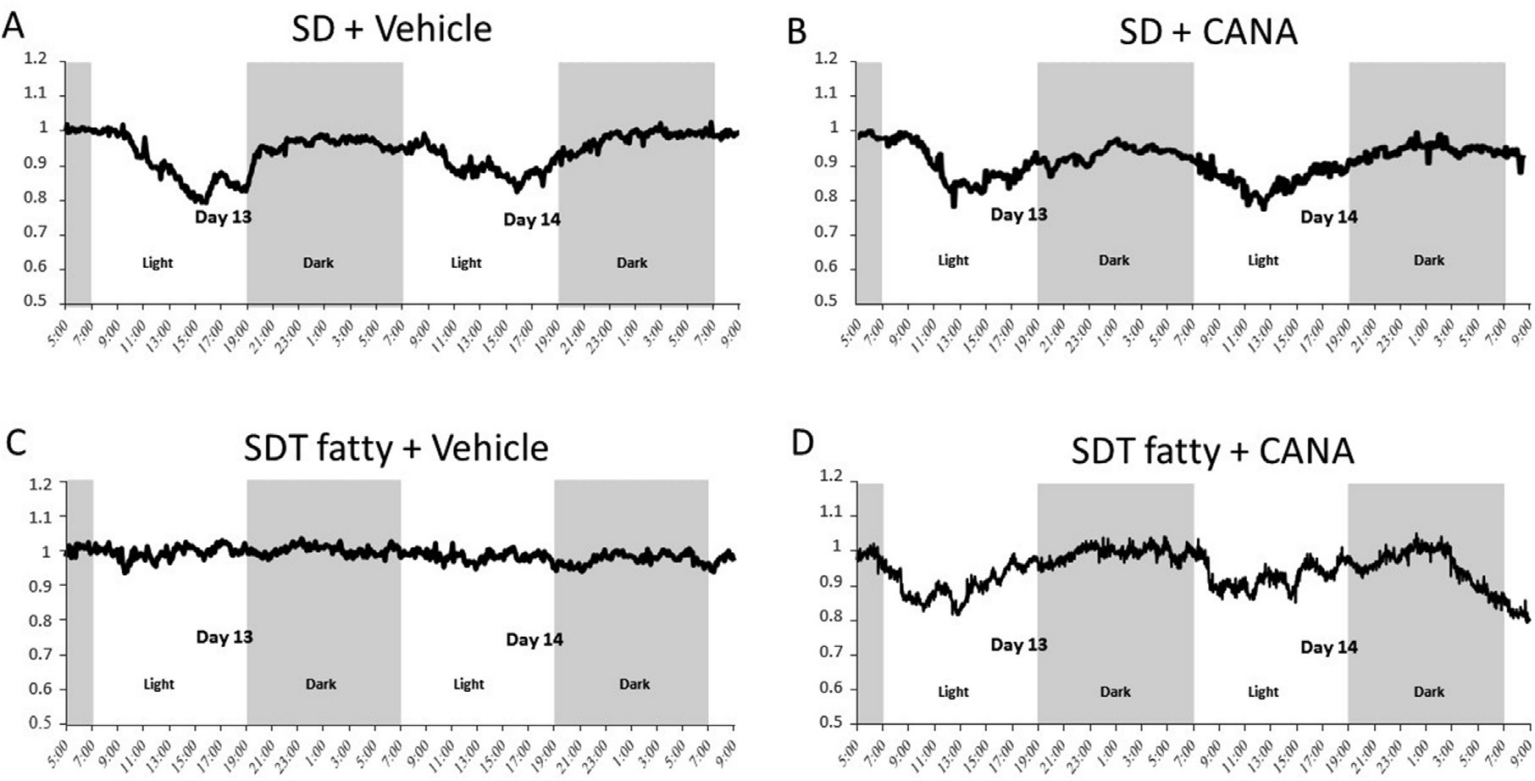
Respiratory quotient after the administration of canagliflozin for 14 days. Data were retrieved at day13 and 14. A; vehicle-treated SD rats. B; CANA-treated SD rats. C; vehicle-treated SDT fatty rats. D; CANA-treated SDT fatty rats. Light; light period. Dark; dark period.

**Figure 3:**
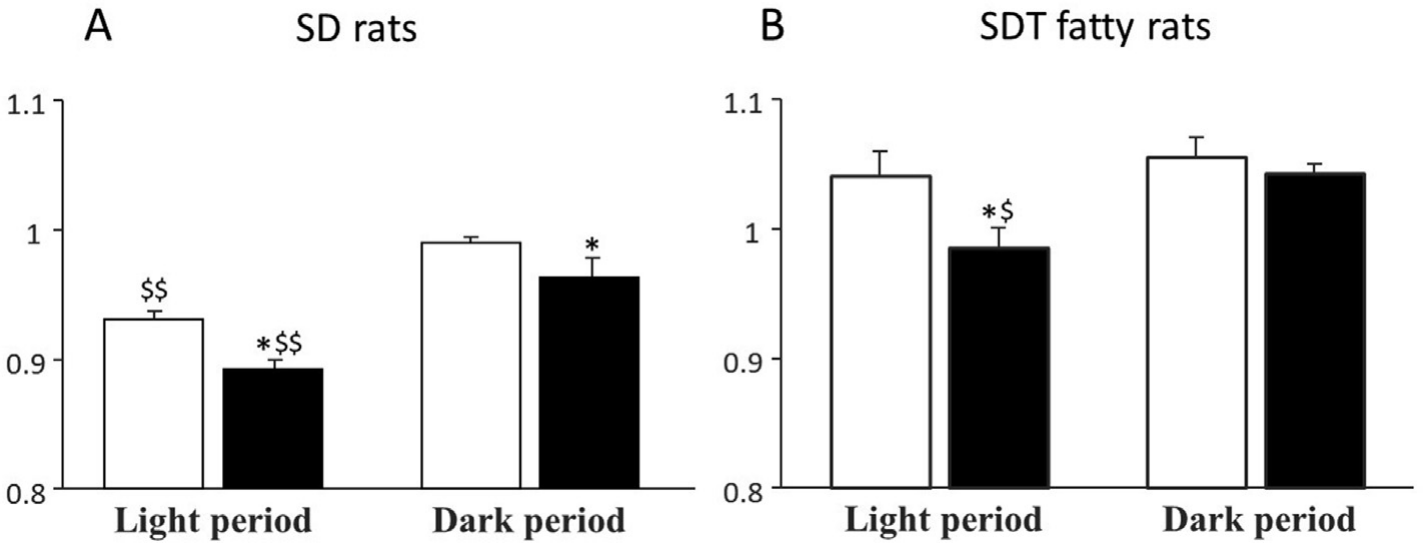
Comparisons of RQs in SD rats and SDT fatty rats. A; SD rats. B SDT fatty rats. Open bars denote RQs in vehicle-treated group and black bars those in CANA-treated group. Data are presented as means ± S.E.M. ∗P < 0.05 vs. vehicle-treated group. ^$^P < 0.05, ^$$^P < 0.01 vs. dark period.

A diurnal rhythm of RQ was clearly observed with its peak during dark period and with its nadir during light period in vehicle-treated SD rats (Figure 2A). The treatment by CANA for 14 days in SD rats didn't affect the diurnal rhythm of RQ (Figure 2B, Figure 3A), but RQ in either light or dark period was significantly lower than that in non-treatment group (Figure 3A). In contrast, RQ was persistently high during dark period and kept still high even in the light period at the same level as in dark period in vehicle-treated SDT fatty rats (Figure 2C). The administration of CANA for 14 days, however, significantly decreased RQ in the light period (Figure 3B) and clearly restored the disrupted diurnal rhythm of RQ observed in SDT fatty rats with a revival of the nadir during light period (Figure 2D), which coincided with a significant increase of blood ketone concentration observed during light period in CANA-treated SDT fatty rats (Figure 1).

### 3.5 The expressions of fat oxidation-related enzymes or molecules in the liver and the white adipose tissue

**Figure 4:**
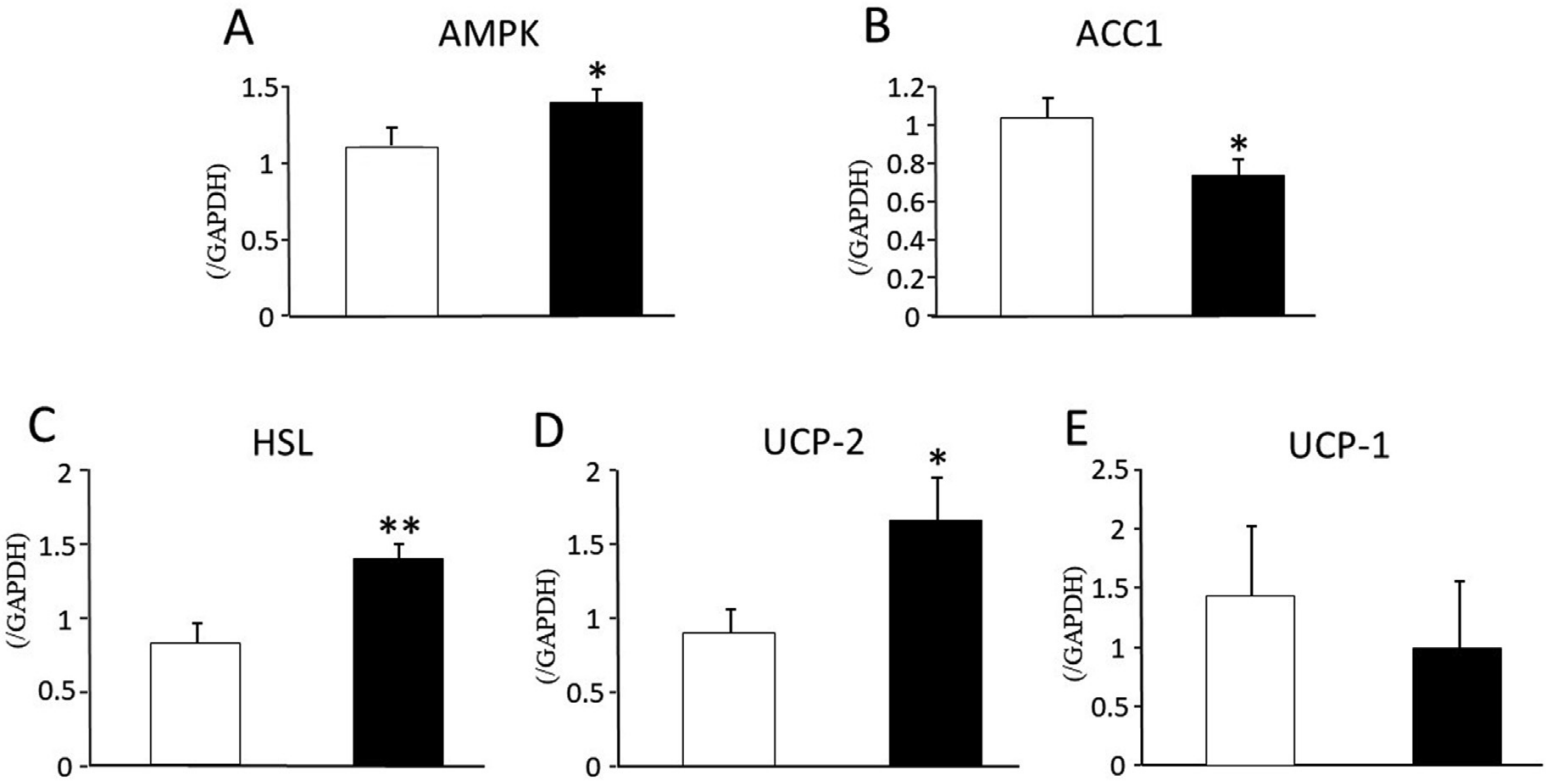
Expressions of fat oxidation-related enzymes in the liver (A, B) and white adipose tissue (C, D, E) after the administration of canagliflozin for 14 days in SDT fatty rats. A; AMPK, B; CPT-1, C; HSL, D; UCP-2, E; UCP-1. Open bars denote data in vehicle-treated group and black bars those in CANA-treated group. Data are presented as means ± S.E.M. ∗P < 0.05, ∗∗P < 0.01 vs. vehicle-treated group.

Quantitative real-time RT-PCR in the liver (Figure 4A and B) revealed a significant increase of AMPK and decrease of ACC-1 expression by the treatment of CANA for 14 days in SDT fatty rats. In the white adipose tissue (Figure 4C, D, E), 14-day CANA treatment in SDT fatty rats brought about a significant increase of HSL and UCP-2 expression with no change of UCP-1 expression.

## 4 Discussion

The major findings of the present study were as follows. First, 14-day administration of CANA to SDT fatty rats brought about a significant decrease of visceral fat volume in spite of comparable body weight and food intake with those in vehicle-treated ones. Secondly, although blood glucose concentration and urine sugar excretion were not significantly affected, bloodβ-ketone concentration was significantly enhanced by CANA treatment during the light period. Thirdly, while SDT fatty rats showed a persistently high RQ level during the whole day, CANA treatment clearly restored the disrupted RQ rhythm with a revival of the nadir during the light period. Finally, RT-PCR revealed the changes of the relevant molecules in the liver and the wthite adipose tissue towards fat oxidation after the administration of CANA for 14 days.

The treatment by SGLT2i including CANA is expected to reduce body weight and body fat amount in human subjects and animal models [11, 12]. The mechanism is attributable not only to the caloric loss into urine but also to the related increase in fat oxidation [11]. Taking the glucose-lowering mechanism such as an enhancement of urinary glucose excretion by SGLT2i into account, the lower renal function could be a negative factor for the decrease of HbA1c level in line with previous reports [5, 13]. It is of interest, however, a significant weight loss was observed even in patients with moderate renal impairment [14], suggesting other mechanisms than glucose excretion into urine might have occurred. Thus, the enhancement of lipolysis after the administration of SGLT2i could be the most likely scenario as a potential mechanism for weight reduction independent of glucose-lowering effect. In our recent report [7], the administration of SGLT2i for 2 years could effectively reduce body weight and fat mass independent of the blood glucose improvement or the renal function in Japanese patients with type 2 diabetes, confirming this hypothesis. In the present study, 14-day administration of CANA to SDT fatty rats didn't affect either blood glucose level or urinary glucose excretion in spite of a significant reduction of visceral fat volume in line with our clinical data, indicating a distinct effect of this agent on body weight or fat amount from blood glucose level. The relatively lower blood glucose level in SDT fatty rats than that in the previous report [8] could be attributable to the fact that the time point of blood sampling was in the latter half of light period (1400–1600) and fasting period might have existed even in hyperphagic SDT fatty rats.

In the SDT fatty rats, blood β-ketone concentration was significantly enhanced by the treatment of CANA for 14 days, especially during the light period compared to vehicle-treated one, suggesting the increase of ketone production reflecting a promotion of fat oxidation during this period. Concomitant with this diurnal rhythm of blood ketone production, 14-day CANA treatment brought about a significant decrease of RQ during the light period. The diurnal rhythm of RQ which had been completely disrupted in SDT fatty rats was clearly revived and comparable to that observed in SD rats. Even in SD rats, 14-day CANA treatment reduced RQ compared to that in non-treated group during either light or dark period, suggesting the increased fat oxidation by the treatment of CANA in non-obese model. Obata et.al [11]. reported that tofogliflozin as SGLT2 inhibitor reduced blood glucose, increased blood ketone body, enhanced fat oxidation-related enzymes and reduced RQ in C57BL/6 mice. It is thus plausible that CANA could enhance a fat oxidation in SD rats in the same way as in SDT fatty rats. The revival of RQ rhythm which coincides with the increase of fat oxidation during the light period suggests the growing out of ‘metabolic inflexibility’ which is often observed in obese diabetic individuals and encompassed with the term of metabolic syndrome [2]. Metabolic syndrome is often associated with the disruption of diurnal rhythm of blood pressure, so called ‘non-dipper’ [15]. It has been reported that SGLT2i improved not only 24-hour blood pressure but also the diurnal rhythm of blood pressure from non-dipper to dipper type in human subjects [16] and rats [17, 18].

Possible mechanisms for this beneficial effect of SGLT2i on the diurnal rhythm of RQ could be attributable to the periodic increase of glucagon/insulin ratio which is often observed after intermittent fasting, gastric bypass and exercise [19, 20, 21]. Increased endogenous glucose production (EGP) following SGLT2i administration has been reported and consistent with this hormonal diversion [22, 23]. The increase in EGP by SGLT2i is consistent with increased gluconeogenesis, because the diversion of oxaloacetate into gluconeogenesis during fasting state inhibits TCA cycle and drive the concomitant generation of ketone bodies from the excess of acetyl CoA concomitant with the increase of fat oxidation [3, 24]. In the present study, by the administration of CANA for 14 days the expressions of relevant molecules changed towards fat oxidation in the liver and adipose tissue which had been retrieved during the light period. These results are quite in line with the previous report in which SGLT2i treatment brought about the acceleration of fat mass reduction and lipolysis accompanied with the enhancement of fat oxidation-related molecules expression such as HSL in the white adipose tissue and carnitine palmitoyl transferase-1 (CPT-1) in the liver [11]. In this aspect, SGLT2i including CANA is expected to reduce body fat amount by the acceleration in β-oxidation of free fatty acid, leading to the increase of β-ketone production, the decrease of RQ during the light period and the correction of ‘metabolic inflexibility’.

This study includes some limitations. First, SDT fatty rats are genetically manipulated animals and the phenotype might be too prominent or severe to be the representative of the obese diabetic model such as diet-induced obese models. It is thus preferable to confirm the reproducibility of the results obtained in this study using another model with milder phenotype. Secondly, our feeding method of CANA using gavage is not a natural way of oral administration and might have put some stress on rats even if vehicle-treated control rats were fed in the same stressful way. Longer-term experiments in which rats are fed the diet mixed with CANA is expected. Thirdly, in SDT fatty rats, there was no effect of CANA on body weight, but there was a significant reduction in visceral fat. The trend of effects of CANA on body weight, subcutaneous and visceral fat area are almost in the same line. Based on the difference between the two measurements, just weighing and estimating fat volume from the CT values within just one vertebra, there is a possibility for a small difference to take place between the measurements. In particular, it is worthwhile to document that the reduction of visceral fat volume was more dominant than that of subcutaneous fat from the aspect of metabolic syndrome. Finally, although enhanced catabolism during the light period could drive the improvement in metabolic health, so-called metabolic flexibility, it still remains uncertain why these adequate catabolic periods are interspersed within the inevitable anabolic periods by the administration of SGLT2i. Precise mechanisms for this issue are to be clarified in future investigations.

In conclusion, CANA as a SGLT2i reduced visceral fat amount via the enhancement of fat oxidation during the light period, leading to an amelioration of metabolic inflexibility in an obese diabetic model. A novel mechanism of CANA prompts the possibility that this new class of anti-diabetic agent could be a promising anti-obesity agent as well.

## Declarations

### Author contribution statement

Kento Hara: Conceived and designed the experiments; Performed the experiments; Analyzed and interpreted the data; Wrote the paper.

Yusuke Sakai: Performed the experiments; Contributed reagents, materials, analysis tools or data.

Yuji Tajiri: Conceived and designed the experiments; Analyzed and interpreted the data; Wrote the paper.

Masatoshi Nomura: Analyzed and interpreted the data.

### Funding statement

This research did not receive any specific grant from funding agencies in the public, commercial, or not-for-profit sectors.

### Data availability statement

Data will be made available on request.

### Declaration of interests statement

This work was supported in part by Mitsubishi Tanabe Pharma Corporation (Osaka, Japan).

### Additional information

No additional information is available for this paper.
